# Human Leukocyte Antigen Polymorphism and Blood Biomarker Profiles in Parkinson’s Disease: A Pilot Study in a Latvian Cohort

**DOI:** 10.3390/biomedicines12122709

**Published:** 2024-11-27

**Authors:** Olga Minibajeva, Guntis Karelis, Maksims Zolovs, Viktorija Ķēniņa

**Affiliations:** 1 Department of Doctoral Studies, Rīga Stradiņš University, LV-1007 Riga, Latvia; 2 Department of Neurology and Neurosurgery, Riga East University Hospital, LV-1079 Riga, Latvia; 3 Department of Infectology, Rīga Stradiņš University, LV-1006 Riga, Latvia; 4 Statistics Unit, Rīga Stradiņš University, LV-1048 Riga, Latvia; 5 Institute of Life Sciences and Technology, Daugavpils University, LV-5401 Daugavpils, Latvia; 6 Department of Neurology, Pauls Stradiņš Clinical University Hospital, LV-1002 Riga, Latvia; 7 Department of Biology and Microbiology, Rīga Stradiņš University, LV-1007 Riga, Latvia; 8 Institute of Oncology and Molecular Genetics, Rīga Stradiņš University, LV-1002 Riga, Latvia

**Keywords:** Parkinson’s disease, HLA, neurofilament light chain, S100 calcium-binding protein A9, kynurenic acid, glutamate decarboxylase (GAD1)

## Abstract

**Background**: Parkinson’s disease (PD) is a neurodegenerative disorder characterised by a high prevalence of sporadic cases. Various molecular mechanisms are involved in its pathogenesis. This pilot study aimed to identify potential risk and protective human leukocyte antigen (HLA) alleles in PD, discover candidate alleles for further research, and evaluate potential blood biomarkers. **Methods**: A total of 43 PD patients and 79 unrelated sex-matched controls were enrolled in this study. We analysed the polymorphism of *HLA-DRB1*, *HLA-DQA1*, and *HLA-DQB1* alleles and the blood levels of biomarkers such as S100 calcium-binding protein A9 (S1000A9), kynurenic acid (KYNA), neurofilament light chain (NfL), and glutamate decarboxylase (GAD1). **Results**: We found that the frequencies of the HLA-DRB1*04, -DQA1*02:01, and -DQA1*03:01 alleles were significantly higher in the PD patients than in the controls, suggesting that these alleles are potential risk factors. Furthermore, the HLA-DQA1*02:01 allele was detected more frequently in the PD group when the disease onset was at 60 years or older. On the contrary, the HLA-DRB1*01 and HLA-DQA1*05:01 alleles were less common in the PD patients, indicating a possible protective effect. Regarding biomarkers, the blood levels of S100 calcium-binding protein A9 were significantly higher, and the kynurenic acid levels were significantly lower in the PD group. The NfL levels were also higher in the PD group but did not reach statistical significance, possibly due to the sensitivity limitations of the ELISA method used. The GAD1 levels showed no significant differences between the two groups. **Conclusions**: Our findings indicate that the HLA-DRB1*01 and -DRB1*04 alleles and the HLA-DQA1*02:01, -DQA1*03:01, and -DQA1*05:01 alleles are associated with PD. Moreover, S100 calcium-binding protein A9 and kynurenic acid can be considered potential blood biomarkers for PD. These findings contribute to the growing body of knowledge on PD and offer new directions for further research in Latvian cohorts.

## 1. Introduction

Human leukocyte antigen (HLA) is crucial for the functioning of the immune system and has significant implications for neurodegenerative disorders. These disorders, including Alzheimer’s disease (AD), Parkinson’s disease (PD), and amyotrophic lateral sclerosis (ALS), are characterised by progressive neuronal damage and loss. HLA class II loci that encode proteins are essential for antigen presentation. They are expressed in microglial cells, which are involved in both phagocytising pathological protein deposits and producing proinflammatory factors that accelerate neuronal death. This dual role underscores the importance of HLA in neurodegeneration [1]. The genetic overlap between neurodegenerative disorders suggests that HLA regions are common genetic risk factors for neurodegenerative disorders. This highlights the shared genetic aetiology and the role of HLA in these diseases [2].

PD is the second most common neurodegenerative disorder after Alzheimer’s disease, characterised by a high prevalence of sporadic cases [3,4]. The risk of developing PD is higher in men than in women [5]. The prevalence of PD rises with age, with a significant increase above the age of 60 years [6]. The main motor clinical symptoms of Parkinson’s disease include bradykinesia, rigidity, and tremor at rest. PD also has non-motor manifestations that may develop years before the classic motor symptoms. These include sleep disturbances, autonomic dysfunction (constipation and urgency to urinate during the day), hyposmia, or psychiatric symptoms such as depression and anxiety [7].

The aetiology of PD is multifactorial, involving environmental and genetic factors [8]. PD is neuropathologically characterised by the loss of nigrostriatal dopaminergic neurons in the substantia nigra and the formation of intracellular Lewy body inclusions. However, neurodegeneration also affects cells in other regions of the neural network [9]. Various molecular mechanisms are involved in the pathogenesis of PD, such as proteostasis disruption, mitochondrial dysfunction, oxidative stress, calcium homeostasis dysregulation, and axonal transport alterations [10,11].

The association of specific HLA alleles with PD can vary significantly across different populations, highlighting the importance of considering ethnic and geographic diversity in genetic studies. Protective and risk HLA alleles show variations that are specific to different populations and not universally consistent [12]. However, in the Baltic countries, including Estonia, Latvia, and Lithuania, there is a notable lack of studies examining the association between HLA alleles and PD. Studies in these countries have primarily focused on the broader epidemiological and clinical aspects of neurodegenerative diseases rather than detailed genetic associations. This lack of research means that there is limited information on HLA alleles conferring an increased risk or protection against PD in Baltic populations. This knowledge underscores the need for more comprehensive genetic studies in these regions to identify potential population-specific genetic markers for PD.

Our pilot study aimed to identify potential protective and risk alleles for PD in a Latvian cohort and to identify candidate alleles for future research. Recent and ongoing research has also focused on identifying biomarkers that facilitate the diagnosis of PD, assess disease severity, and predict disease progression. The diversity of pathogenetic mechanisms of the disease requires the selection of multiple biomarkers. We performed extensive biomarker testing that included neurofilament light chain (NfL) as a promising biomarker of neurodegeneration [13], S100 calcium-binding protein A9 (S100A9) and kynurenic acid (KYNA) as potential blood-based biomarkers of immune system disruption [14,15], and glutamate decarboxylase (GAD1) as a marker of GABAergic system impairment [16].

## 2. Materials and Methods

### 2.1. Study Population

**Figure 1:**
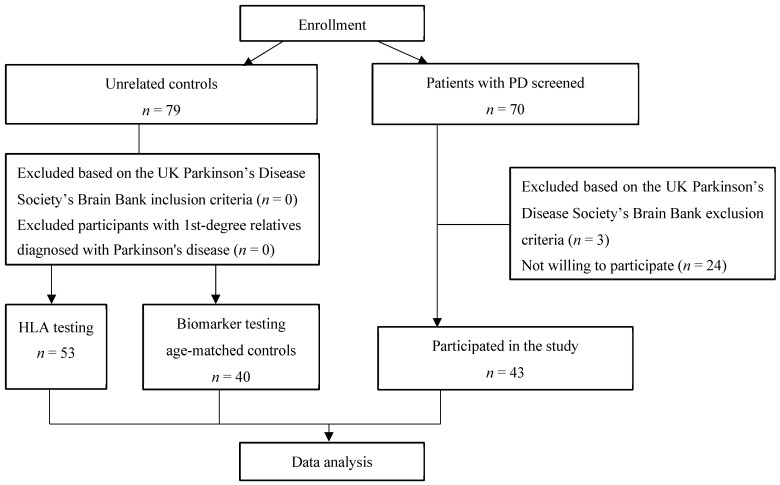
Flowchart of this study.

For this study, 43 PD patients and 79 unrelated, sex-matched healthy controls were enrolled. All the participants were recruited from the Gaiļezers Clinic of Riga East University Hospital, Department of Neurology and Neurosurgery, from 2019 to 2020 (Figure 1). All the PD patients were diagnosed with PD according to the UK Parkinson’s Disease Society Brain Bank criteria [17] and recruited into the study at the time of diagnosis. The control group consisted of individuals without PD who presented to a neurologist for other disorders, and those with first-degree relatives diagnosed with PD were excluded.

Demographic variables, including sex, age, age at disease onset, disease duration, and clinical type, were recorded for all the PD participants. Age at onset was based on medical records and the patients’ recollections of their first symptoms. The worldwide prevalence rates of PD vary by age group [18]. Therefore, for the evaluation of alleles, the PD patients were divided into two groups: (1) those with an onset age <60 years and (2) patients with an onset age ≥60 years. According to the classification of clinical phenotypes, the participants were divided into the tremor-dominant (TD), postural instability/gait difficulty (PIGD), and mixed phenotypes of PD [19]. Professionally trained and qualified neurologists evaluated the patients.

The PD patients were evaluated using the Movement Disorder Society-Sponsored Revision of the Unified Parkinson’s Disease Rating Scale (MDS-UPDRS) during the ON condition. Disease severity was evaluated using the Hoehn and Yahr scale (staging 1–5) [20,21].

The study protocol was approved by the Science Department of the Riga East University Hospital Clinic Gaiļezers and the Central Medical Ethics Committee of Latvia. All participants provided written informed consent. This study adhered to the Declaration of Helsinki on Ethical Principles for Medical Research Involving Human Subjects (2008).

### 2.2. Laboratory Analyses

Peripheral blood samples were taken from the PD patients and unrelated controls. Two 5 mL blood samples were obtained from each participant. The samples were stored at +4 °C and delivered to the laboratory within 2 h.

To detect HLA class II alleles, 5 mL volumes of the peripheral blood samples were placed in tubes with anticoagulant EDTA, then frozen at −20 °C, and stored in the laboratory until testing. The analysis included human DNA extraction from blood and HLA gene typing with a low-resolution polymerase chain reaction. Chromosomal DNA was extracted from leukocytes using a QIAamp^®^ DNA Blood Kit (QIAGEN GmbH, Hilden, Germany). The gene-typing method included the detection of alleles of the *HLA-DRB1*, -*DQA1*, and *-DQB1* genes, using specific primers according to the manufacturer’s instructions (DNA-Technology, Moscow, Russia). Amplification was performed using a programmed thermocycler (DTlite, DNA-Technology).

To detect biomarkers, 5 mL volumes of the peripheral blood samples were placed in tubes with the anticoagulant EDTA and then centrifuged at 4000 rpm for 15 min; the supernatant was aspirated and aliquoted, frozen at −20 °C, and stored in the laboratory until testing.

NfL and KYNA levels were measured in the serum using a commercial enzyme-linked immunosorbent assay (Abbexa Ltd., Cambridge, UK). The method of determination was based on sandwich enzyme-linked immunosorbent assay technology. The measuring range was 15.6 pg/mL–1000 pg/mL for NfL and 2.47 ng/mL to 200 ng/mL for KYNA.

The GAD1 and S1000A9 levels in the serum were measured with a commercial enzyme-linked immunosorbent assay (chemiluminescence) (NOVUS Biologicals, LLC, Centennial, CO, USA). The method of determination was based on the sandwich-CLIA principle. The measuring range was 0.31 ng/mL–20 ng/mL for GAD1 and 0.16 ng/mL to 10 ng/mL for S100A9.

### 2.3. Statistical Analyses

Statistical analyses were performed with SPSS Statistics 28.0 (Chicago, IL, USA) and Jamovi Version 2.5.3. Data distribution was assessed by inspecting a normal Q-Q plot and using the Shapiro–Wilk test. Mean values and standard deviations are used to report normally distributed data, the medians and interquartile ranges (IQRs) are used to summarise non-normally distributed data, and frequencies and percentages are used to describe nominal data. Allele frequency was defined as the number of participants with homozygote or heterozygote allele presentations. Fisher’s exact test was used to examine the frequency distribution between the PD and control groups. The Mann–Whitney test was used to compare the NfL, S100A9, and GAD1 levels, and the independent-samples *t* test was used to compare the kynurenic acid levels between the PD and control groups. Statistical significance was only accepted at *p* < 0.05.

## 3. Results

Of the 43 patients with sporadic PD, 23 (53.5%) were female and 20 (46.5%) were male. The mean age of the patients was 65 years (±8.9 SD), and the average disease duration was 6 years. The most common comorbidities were arterial hypertension and thyroid gland disorders (hypothyroidism and hyperthyroidism), followed by cancer and diabetes mellitus. Among the patients with oncological diseases, none had brain cancer. The demographic and clinical characteristics of the PD group are presented in Table 1.

We extensively analysed HLA polymorphism, including 12 *HLA-DRB1* alleles, 7 *HLA-DQA1* alleles, and 10 *HLA-DQB1* alleles.

Of all 12 *HLA-DRB1* alleles identified in the PD group, 7 occurred at a frequency ≥10%. The most commonly observed HLA-DRB1*04 (37.2%) and -DRB1*07 (37.2%) alleles were followed by -DRB1*01 (23.3%), -DRB1*15 (23.3%), -DRB1*11 (20.9%), -DRB1*13 (18.6%), and -DRB1*17 (16.3%). We found that the PD patients presented a statistically significant higher frequency of the HLA-DRB1*04 allele than controls (*p* < 0.001). By contrast, HLA-DRB1*01 was found at a lower frequency in the PD patients than in the controls (*p* = 0.032). The statistical analysis of HLA-DRB1*04 showed a wide confidence interval, which may affect the reliability of the results. Moreover, we excluded the HLA-DRB1*08 allele from further investigation because its confidence interval crossed 1.

Among the *HLA-DQA1* alleles in the PD group, almost all were observed at a frequency greater than 10%. A statistically significant difference between groups was observed only for the HLA-DQA1*01:03 (*p* < 0.001), -DQA1*02:01 (*p* = 0.03), -DQA1*03:01 (*p* = 0.003), and -DQA1*05:01 alleles (*p* = 0.023). An analysis of the HLA-DQA1*01:03 allele revealed a vast confidence interval; therefore, we excluded it from further evaluation.

Of the ten *HLA-DQB1* alleles identified, frequencies ≥ 10% were observed for HLA-DQB1*02:01:02, -DQB1*03:01, -DQB1*03:03, -DQB1*05:01, -DQB1*05:02:04, and -DQB1*06:02:08. There were no statistically significant differences between the PD and control groups (*p* > 0.05). We excluded the HLA-DQB1*03:04 allele because its confidence interval crossed 1. The frequencies of the HLA class II alleles are summarised in Table 2.

Alleles with statistically significant differences between the PD group and controls were further investigated. We analysed the frequency of allele polymorphism according to age at disease onset. All the PD patients were divided into two subgroups (age < 60 and age ≥ 60). The HLA-DQA1*02:01 allele was statistically significantly more frequent in the PD group with disease onset at 60 years or older (*p* = 0.033). An analysis of the other alleles did not show statistically significant differences in the PD subgroups < 60 years old and 60 years or older (*p* > 0.05). The frequencies of HLA-DRB1*01, *04 and HLA-DQA1*02:01, *03:01, and *05:01 allele polymorphism in the PD subgroups are shown in Table 3.

We assessed the levels of blood biomarkers in all patients in the PD group and in 40 age- and sex-matched participants in the control group. NfL levels were higher in the PD group, but the difference was not statistically significant (*p* > 0.05). GAD1 levels were similar in the PD patients and their age-matched controls. The KYNA levels in the PD group were statistically significantly lower than those in the controls (*p* = 0.005). Furthermore, S100A9 levels were statistically significantly higher in the PD patients than in the controls (*p* = 0.005). The levels of the blood biomarkers analysed in the PD patients and age-matched controls are shown in Table 4.

## 4. Discussion

Our pilot study aimed to identify alleles that could potentially serve as protective or risk factors for PD in a Latvian cohort and to pinpoint candidate alleles for further research. We provided a detailed characterisation of our cohort, including an analysis of biomarkers such as NfL, S100A9, kynurenic acid (KYNA), and GAD1, all of which are well documented in the literature for their importance in neurodegenerative diseases [13,14,15].

The HLA loci are the most polymorphic in the human genome. The association of HLA polymorphism with PD was first reported more than ten years ago [22]. The accumulation of the protein α-synuclein is accompanied by reactive microgliosis, increased expression of inflammatory cytokines, and T cell activation, and its reactivity may depend on HLA allele variations [23]. Previous studies have found an association between the *HLA-DRB1*, *-DQA1*, and *-DQB1* loci and PD [24,25], and polymorphisms at the *HLA-DRB1* locus have most often been studied.

In this current study, we thoroughly analysed the HLA region and examined its association with PD in a Latvian cohort. We found that the PD patients in our cohort had a statistically significantly higher frequency of the HLA-DRB1*04 allele, suggesting that it is a potential risk factor for PD. Our results are in contrast to those of several studies where the HLA-DRB1*04 allele was shown to play a protective role [22,24,26,27,28,29]. This discrepancy with the data of previous studies may also be due to the small number of patients in our cohort. However, some studies suggest that the HLA-DRB1*04 allele has a risk association [25] and that different subtypes may have different positive or negative associations with PD [12]. Moreover, HLA-DRB1*01 was statistically significantly less frequently detected in the PD patient group in this current study. Unlike several previous studies that either did not find an association between the HLA-DRB1*01 allele and PD [27] or showed an association between the HLA-DRB1*01:01 allele subtype and an increased risk of PD [30], our study observed a possible protective role for this allele. Previous investigations provide conflicting data on the significance of this allele, so our findings may be relevant to our region. This discrepancy underscores the importance of considering ethnic and geographic diversity in genetic studies.

This study found statistically significant differences between the PD group and controls when evaluating allelic polymorphism at the *HLA-DQA1* locus. Both HLA-DQA1*03:01 and HLA-DQA1*02:01 were statistically more prevalent in the PD group. In addition, HLA-DQA*02:01 was statistically significantly more frequently detected in the PD group with disease onset at 60 years or older. Ageing is a well-known risk factor for PD [31]. Our findings indicate that in addition to the mechanisms underlying ageing, the involvement of the immune system may also play a role in neurodegeneration. The association of the HLA-DQA1*02:01 allele with PD has not been widely discussed in previous studies. The results of our research suggest that HLA-DQA1*02:01 is a risk allele and may have a specific association with late-onset PD, warranting further investigation. An association between the HLA-DQA1*03:01 allele and PD was observed in previous studies; however, in contrast to the results of our research, negative associations were obtained in these studies [28,29]. At the same time, HLA-DQA*05:01 was statistically significantly more frequently detected in the control group. The association of the HLA DQA1*05:01 allele with PD has not been widely discussed in previous studies, so more research is needed to evaluate the protective effect against the disease in the Latvian cohort. This current study highlights the potential role of *HLA-DQA1* alleles polymorphism in the pathogenesis of PD.

The role of S100A9 in the pathogenesis of PD has been extensively studied. Histologically, S100A9 has been found in the brain tissue of patients with PD in co-aggregation with α-synuclein [32]. Regarding blood biomarkers, our study showed that S100 calcium-binding protein A9 levels were statistically significantly higher in the PD patients than in the controls, indicating its potential as a biomarker for PD. Elevated levels of S100A9 have been associated with neuroinflammation, and this increase has been implicated in the pathogenesis of neurodegenerative diseases [33]. This finding supports previous research suggesting the involvement of S100A9 in PD. Overall, our results highlight the potential of S100A9 as a blood biomarker and provide further evidence of its potential involvement in the inflammatory processes associated with PD.

The potential effects of the metabolites of the kynurenine pathway on neuroinflammation in PD are mainly based on the neurotoxic role of quinolinic acid and the neuroprotective role of kynurenic acid in the brain [34]. In our study, the KYNA levels were statistically significantly lower in the PD group than in the controls. KYNA is a neuroactive metabolite of the kynurenine pathway, and it has been implicated in neuroprotection [14,35,36]. The reduced kynurenic acid levels in the PD patients may reflect the dysregulation of this pathway and contribute to neurodegenerative processes. This observation aligns with that of other studies that reported alterations in the kynurenine pathway in PD [37,38].

GAD1 is an essential enzyme for the conversion of glutamate to gamma-aminobutyric acid (GABA), a key inhibitory neurotransmitter in the brain [39,40]. GABA has also been identified as an important gastrointestinal neurotransmitter that is potentially involved in both secretory and motor gastrointestinal functions [41]. Although research indicates that changes in the GABAergic system are involved in PD, our study did not find significant differences in GAD1 blood levels between the PD patients and controls, suggesting that it may not be a reliable biomarker for the disease. While this parameter alone may not be a definitive marker, its role within the broader GABAergic system warrants further investigation to elucidate its potential utility in diagnosing and monitoring PD.

NfL is a widely studied biomarker for PD and other neurological disorders. It is a blood biomarker indicating neuroaxonal damage. Previous research indicates that the blood levels of NfL are higher in PD patients than in controls [13,42]. We investigated the NfL levels in patients with PD and age-matched controls. However, although the NfL levels were higher in the PD group, they did not show a statistically significant difference. One potential reason for the lack of statistical significance is the sensitivity of the ELISA method used. Although widely used, an ELISA may not be as sensitive as other methods, such as a single-molecule array (Simoa) or electrochemiluminescence immunoassays. These alternative methods have been shown to detect NfL at lower concentrations and with higher accuracy [43]. The ELISA method has a higher limit of detection than more sensitive assays such as Simoa, which can measure NfL levels at subpicogram per millilitre concentrations, providing more precise and reliable measurements [44].

Our pilot study provides valuable insights into PD’s genetic and biomarker profiles in a Latvian cohort, identifies potential risk and protective HLA alleles for PD, and highlights the relevance of certain blood biomarkers. These findings contribute to the growing body of knowledge on PD genetics and emphasise the importance of considering population-specific variations.

Despite this study’s inclusion of both genetic predisposition and potential blood biomarkers for disease, it has several limitations. First, the small number of participants restricted the statistical analysis, resulting in some ambiguous findings. Second, for some of the testing methods discussed in the results, we could not employ more precise techniques, which may have impacted the accuracy of the findings. More sensitive methods, such as Simoa, are recommended for determining the level of NfL in the blood. Further studies with a larger number of patients and more centres are needed to evaluate HLA alleles in the Latvian population.

However, the strengths of this study are that it estimates the prevalence of alleles among patients with PD and highlights potential risk and protective alleles. Additionally, it is the first study to examine the HLA II class alleles and blood biomarkers in PD patients in Latvia.

## 5. Conclusions

This study investigated the relationship between HLA II class alleles, potential blood biomarkers, and PD. The findings demonstrate that the HLA-DRB1*01 and -DRB1*04 alleles and HLA-DQA1*02:01, -DQA1*03:01, and -DQA1*05:01 alleles are associated with PD. Furthermore, this study provides additional evidence that S100A9 and KYNA can be considered potential blood biomarkers for PD. Despite some limitations, the findings of this study improve our understanding of the genetic factors underlying PD in a Latvian cohort and offer new ideas for further research. Additionally, more research is required to validate our results, including confirmation in a larger sample size and evaluation of potential biomarkers with more sensitive techniques.

## Figures and Tables

**Table 1:** Demographic and clinical characteristics of Parkinson’s disease (PD) patients.

Demographics and Clinical Characteristics	Total (*n* = 43)
Age (years), mean ± SD	65.2 ± 8.94
Age at onset, median (Q1–Q3)	59 (49–64)
Gender, *n* (%)	
Male	20 (46.5%)
Female	23 (53.5%)
Duration of PD (years), median (Q1–Q3)	6 (4–10)
Hypertension, *n* (%)	18 (41.9%)
Disorders of the thyroid gland, *n* (%)	15 (34.9%)
Cancer, *n* (%)	4 (9.3%)
Diabetes mellitus, *n* (%)	3 (7%)
Clinical subgroups, *n* (%)	
Tremor-dominant	14 (32.6%)
PIGD	25 (58.1%)
Mixed	4 (9.3%)
Hoehn and Yahr stage, *n* (%)	
HY—1	17 (39.5%)
HY—2	15 (34.9%)
HY—3	11 (25.6%)

Abbreviation: HY = Hoehn and Yahr stage; PIGD = postural instability/gait difficulty.

**Table 2:** Frequency of *HLA-DRB1*, *-DQA1*, and *-DQB1* allele polymorphism in PD patients and controls.

HLA Alleles	Controls	PD	OR (95% CI)	*p*-Value
	*n* = 53	*n* = 43		
DRB1				
01	24 (45.3%)	10 (23.3%)	0.366 (0.15–0.892)	0.032
04	3 (5.7%)	16 (37.2%)	9.88 (2.64–36.9)	<0.001
07	10 (18.9%)	16 (37.2%)	2.55 (1.01–6.43)	0.064
08	7 (13.2%)	0	0.0713 (0.003–1.29)	0.016
09	1 (1.9%)	0	0.402 (0.016–10.1)	1.0
11	12 (22.6%)	9 (20.9%)	0.904 (0.341–2.4)	1.0
12	1 (1.9%)	4 (9.3%)	5.33 (0.573–49.6)	0.17
13	18 (34%)	8 (18.6%)	0.444 (0.171–1.16)	0.11
14	7 (13.2%)	3 (7%)	0.493 (0.119–2.03)	0.504
15	5 (9.4%)	10 (23.3%)	2.91 (0.911–9.29)	0.09
16	8 (15.1%)	2 (4.7%)	0.274 (0.055–1.37)	0.177
17	6 (11.3%)	7 (16.3%)	1.52 (0.471–4.93)	0.556
DQA1				
01:01	15 (28.3%)	7 (16.3%)	0.493 (0.18–1.35)	0.223
01:02	23 (43.4%)	14 (32.6%)	0.63 (0.272–1.46)	0.3
01:03	0	9 (20.9%)	29.5 (1.66–523)	<0.001
02:01	12 (22.6%)	19 (44.2%)	2.7 (1.12–6.53)	0.03
03:01	9 (17%)	20 (46.5%)	4.25 (1.67–10.8)	0.003
04:01	1 (1.9%)	3 (7%)	3.9 (0.391–38.9)	0.322
05:01	29 (54.7%)	13 (30.2%)	0.359 (0.154–0.836)	0.023
DQB1				
02:01:02	16 (30.2%)	21 (48.8%)	2.21 (0.955–5.1)	0.091
03:01	23 (43.4%)	12 (27.9%)	0.505 (0.214–1.19)	0.139
03:02	5 (9.4%)	3 (7%)	0.72 (0.162–3.2)	0.727
03:03	5 (9.4%)	6 (14%)	1.56 (0.441–5.5)	0.534
03:04	0	4 (9.3%)	12.2 (0.638–233)	0.037
04:01	0	1 (2.3%)	3.78 (0.15–95.1)	0.448
04:01:02	3 (5.7%)	1 (2.3%)	0.397 (0.039–3.96)	0.625
05:01	15 (28.3%)	13 (30.2%)	1.1 (0.454–2.66)	1.0
05:02:04	7 (13.2%)	6 (14%)	1.07 (0.33–3.44)	1.0
06:02:08	19 (35.8%)	12 (27.9%)	0.693 (0.29–1.66)	0.511

Abbreviation: PD = Parkinson’s disease; HLA = human leukocyte antigen; OR = odds ratio; 95% CI = 95% confidence interval.

**Table 3:** Frequency of HLA-DRB1*01, -DRB1*04 and HLA-DQA1*02:01, -DQA1*03:01, and -DQA1*05:01 allele polymorphism according to age at disease onset.

HLA Alleles	PD		*p*-Value
	Age < 60 (*n* = 22)	Age ≥ 60 (*n* = 21)	
DRB1			
01	5 (22.7%)	5 (23.8%)	1
04	10 (45.5%)	6 (28.6%)	0.347
DQA1			
02:01	6 (27.3%)	13 (61.9%)	0.033
03:01	12 (54.5%)	8 (38.1%)	0.364
05:01	7 (31.8%)	6 (28.6%)	1

Abbreviation: PD = Parkinson’s disease; HLA = human leukocyte antigen.

**Table 4:** The neurofilament light chain (NfL), S100 calcium-binding protein A9 (S100A9), kynurenic acid (KYNA), and glutamate decarboxylase (GAD1) levels in Parkinson’s disease (PD) patients and age-matched controls.

Blood Biomarkers	Age-Matched Controls *n* = 40	PD *n* = 43	*p*-Value
Age (years), mean ± SD	62 ± 7.3	65.2 ± 8.9	0.079
Gender, male, *n* (%)	19 (47.5%)	20 (46.5%)	1
Neurofilament light chain, pg/mL, median (Q1–Q3)	303.5 (211.25–460.35)	335.3 (255.8–415.5)	0.88
S100 calcium-binding protein A9, ng/mL, median (Q1–Q3)	2.71 (1.1–4.02)	3.51 (2.56–6.04)	0.005
Kynurenic acid, ng/mL, mean ± SD	183.08 ± 9.18	177.4 ± 8.86	0.005
Glutamate decarboxylase (GAD1), ng/mL, median (Q1–Q3)	0.34 (0.0–0.76)	0.37 (0.27–0.67)	0.35

## Data Availability

The data presented in this study are available upon request from the corresponding author. Due to ethical restrictions, they are not publicly available.

## References

[1] Aliseychik M.P., Andreeva T.V., Rogaev E.I. (2018). Immunogenetic Factors of Neurodegenerative Diseases: The Role of HLA Class II. Biochemistry.

[2] Wightman D.P., Savage J.E., Tissink E., Romero C., Jansen I.E., Posthuma D. (2023). The genetic overlap between Alzheimer’s disease, amyotrophic lateral sclerosis, Lewy body dementia, and Parkinson’s disease. Neurobiol. Aging.

[3] de Lau L.M., Breteler M.M. (2006). Epidemiology of Parkinson’s disease. Lancet Neurol..

[4] Elbaz A., Carcaillon L., Kab S., Moisan F. (2016). Epidemiology of Parkinson’s disease. Rev. Neurol..

[5] Baldereschi M., Di Carlo A., Rocca W.A., Vanni P., Maggi S., Perissinotto E., Grigoletto F., Amaducci L., Inzitari D. (2000). Parkinson’s disease and parkinsonism in a longitudinal study: Two-fold higher incidence in men. ILSA Working Group. Italian Longitudinal Study on Aging. Neurology.

[6] Pringsheim T., Jette N., Frolkis A., Steeves T.D. (2014). The prevalence of Parkinson’s disease: A systematic review and meta-analysis. Mov. Disord..

[7] Postuma R.B., Berg D., Stern M., Poewe W., Olanow C.W., Oertel W., Obeso J., Marek K., Litvan I., Lang A.E. (2015). MDS clinical diagnostic criteria for Parkinson’s disease. Mov. Disord..

[8] Simon D.K., Tanner C.M., Brundin P. (2020). Parkinson Disease Epidemiology, Pathology, Genetics, and Pathophysiology. Clin. Geriatr. Med..

[9] Gibb W.R., Lees A.J. (1988). The relevance of the Lewy body to the pathogenesis of idiopathic Parkinson’s disease. J. Neurol. Neurosurg. Psychiatry.

[10] Du X.Y., Xie X.X., Liu R.T. (2020). The Role of α-Synuclein Oligomers in Parkinson’s Disease. Int. J. Mol. Sci..

[11] Poewe W., Seppi K., Tanner C.M., Halliday G.M., Brundin P., Volkmann J., Schrag A.E., Lang A.E. (2017). Parkinson disease. Nat. Rev. Dis. Primers.

[12] Misra M.K., Damotte V., Hollenbach J.A. (2018). The immunogenetics of neurological disease. Immunology.

[13] Mollenhauer B., Dakna M., Kruse N., Galasko D., Foroud T., Zetterberg H., Schade S., Gera R.G., Wang W., Gao F. (2020). Validation of Serum Neurofilament Light Chain as a Biomarker of Parkinson’s Disease Progression. Mov. Disord..

[14] Ostapiuk A., Urbanska E.M. (2022). Kynurenic acid in neurodegenerative disorders-unique neuroprotection or double-edged sword?. CNS Neurosci. Ther..

[15] Toleikis Z., Ziaunys M., Baranauskiene L., Petrauskas V., Jaudzems K., Smirnovas V. (2021). S100A9 Alters the Pathway of Alpha-Synuclein Amyloid Aggregation. Int. J. Mol. Sci..

[16] Alharbi B., Al-Kuraishy H.M., Al-Gareeb A.I., Elekhnawy E., Alharbi H., Alexiou A., Papadakis M., Batiha G.E.-S. (2024). Role of GABA pathway in motor and non-motor symptoms in Parkinson’s disease: A bidirectional circuit. Eur. J. Med. Res..

[17] Hughes A.J., Daniel S.E., Kilford L., Lees A.J. (1992). Accuracy of clinical diagnosis of idiopathic Parkinson’s disease: A clinico-pathological study of 100 cases. J. Neurol. Neurosurg. Psychiatry.

[18] Zhu J., Cui Y., Zhang J., Yan R., Su D., Zhao D., Wang A., Feng T. (2024). Temporal trends in the prevalence of Parkinson’s disease from 1980 to 2023: A systematic review and meta-analysis. Lancet Healthy Longev..

[19] Stebbins G.T., Goetz C.G., Burn D.J., Jankovic J., Khoo T.K., Tilley B.C. (2013). How to identify tremor dominant and postural instability/gait difficulty groups with the movement disorder society unified Parkinson’s disease rating scale: Comparison with the unified Parkinson’s disease rating scale. Mov. Disord..

[20] Fahn S., Elton R.L., Members U.P., Fahn S., Marsden C.D., Calne D., Goldstein M. (1987). Unified Parkinson’s disease rating scale. Recent Developments in Parkinson’s Disease.

[21] Goetz C.G., Tilley B.C., Shaftman S.R., Stebbins G.T., Fahn S., Martinez-Martin P., Poewe W., Sampaio C., Stern M.B., Dodel R. (2008). Movement Disorder Society-sponsored revision of the Unified Parkinson’s Disease Rating Scale (MDS-UPDRS): Scale presentation and clinimetric testing results. Mov. Disord..

[22] Naito T., Satake W., Ogawa K., Suzuki K., Hirata J., Foo J.N., Tan E.K., Toda T., Okada Y. (2021). Trans-Ethnic Fine-Mapping of the Major Histocompatibility Complex Region Linked to Parkinson’s Disease. Mov. Disord..

[23] Sulzer D., Alcalay R.N., Garretti F., Cote L., Kanter E., Agin-Liebes J., Liong C., McMurtrey C., Hildebrand W.H., Mao X. (2017). T cells from patients with Parkinson’s disease recognize α-synuclein peptides. Nature.

[24] Ahmed I., Tamouza R., Delord M., Krishnamoorthy R., Tzourio C., Mulot C., Nacfer M., Lambert J.C., Beaune P., Laurent-Puig P. (2012). Association between Parkinson’s disease and the *HLA-DRB1* locus. Mov. Disord..

[25] Pandi S., Chinniah R., Sevak V., Ravi P.M., Raju M., Vellaiappan N.A., Karuppiah B. (2021). Association of *HLA-DRB1*, *DQA1* and *DQB1* alleles and haplotype in Parkinson’s disease from South India. Neurosci. Lett..

[26] Le Guen Y., Luo G., Ambati A., Damotte V., Jansen I., Yu E., Nicolas A., de Rojas I., Peixoto Leal T., Miyashita A. (2023). Multiancestry analysis of the HLA locus in Alzheimer’s and Parkinson’s diseases uncovers a shared adaptive immune response mediated by. Proc. Natl. Acad. Sci. USA.

[27] Sun C., Wei L., Luo F., Li Y., Li J., Zhu F., Kang P., Xu R., Xiao L., Liu Z. (2012). *HLA-DRB1* alleles are associated with the susceptibility to sporadic Parkinson’s disease in Chinese Han population. PLoS ONE.

[28] Wissemann W.T., Hill-Burns E.M., Zabetian C.P., Factor S.A., Patsopoulos N., Hoglund B., Holcomb C., Donahue R.J., Thomson G., Erlich H. (2013). Association of Parkinson disease with structural and regulatory variants in the HLA region. Am. J. Hum. Genet..

[29] Yu E., Ambati A., Andersen M.S., Krohn L., Estiar M.A., Saini P., Senkevich K., Sosero Y.L., Sreelatha A.A.K., Ruskey J.A. (2021). Fine mapping of the HLA locus in Parkinson’s disease in Europeans. npj Parkinson’s Dis..

[30] Hollenbach J.A., Norman P.J., Creary L.E., Damotte V., Montero-Martin G., Caillier S., Anderson K.M., Misra M.K., Nemat-Gorgani N., Osoegawa K. (2019). A specific amino acid motif of. Proc. Natl. Acad. Sci. USA.

[31] Pang S.Y., Ho P.W., Liu H.F., Leung C.T., Li L., Chang E.E.S., Ramsden D.B., Ho S.L. (2019). The interplay of aging, genetics and environmental factors in the pathogenesis of Parkinson’s disease. Transl. Neurodegener..

[32] Horvath I., Iashchishyn I.A., Moskalenko R.A., Wang C., Wärmländer S.K.T.S., Wallin C., Gräslund A., Kovacs G.G., Morozova-Roche L.A. (2018). Co-aggregation of pro-inflammatory S100A9 with α-synuclein in Parkinson’s disease: Ex vivo and in vitro studies. J. Neuroinflamm..

[33] Wang S., Song R., Wang Z., Jing Z., Ma J. (2018). S100A8/A9 in Inflammation. Front. Immunol..

[34] Venkatesan D., Iyer M., Narayanasamy A., Siva K., Vellingiri B. (2020). Kynurenine pathway in Parkinson’s disease—An update. eNeurologicalSci.

[35] Fathi M., Vakili K., Yaghoobpoor S., Tavasol A., Jazi K., Hajibeygi R., Shool S., Sodeifian F., Klegeris A., McElhinney A. (2022). Dynamic changes in metabolites of the kynurenine pathway in Alzheimer’s disease, Parkinson’s disease, and Huntington’s disease: A systematic Review and meta-analysis. Front. Immunol..

[36] Heilman P.L., Wang E.W., Lewis M.M., Krzyzanowski S., Capan C.D., Burmeister A.R., Du G., Escobar Galvis M.L., Brundin P., Huang X. (2020). Tryptophan Metabolites Are Associated with Symptoms and Nigral Pathology in Parkinson’s Disease. Mov. Disord..

[37] Chang K.H., Cheng M.L., Tang H.Y., Huang C.Y., Wu Y.R., Chen C.M. (2018). Alternations of Metabolic Profile and Kynurenine Metabolism in the Plasma of Parkinson’s Disease. Mol. Neurobiol..

[38] Holmans P., Moskvina V., Jones L., Sharma M., Vedernikov A., Buchel F., Saad M., Bras J.M., Bettella F., Nicolaou N. (2013). A pathway-based analysis provides additional support for an immune-related genetic susceptibility to Parkinson’s disease. Hum. Mol. Genet..

[39] Murueta-Goyena A., Andikoetxea A., Gómez-Esteban J.C., Gabilondo I. (2019). Contribution of the GABAergic System to Non-Motor Manifestations in Premotor and Early Stages of Parkinson’s Disease. Front. Pharmacol..

[40] Terkelsen M.H., Hvingelby V.S., Pavese N. (2022). Molecular Imaging of the GABAergic System in Parkinson’s Disease and Atypical Parkinsonisms. Curr. Neurol. Neurosci. Rep..

[41] Auteri M., Zizzo M.G., Serio R. (2015). GABA and GABA receptors in the gastrointestinal tract: From motility to inflammation. Pharmacol. Res..

[42] Chen C.H., Lee B.C., Lin C.H. (2020). Integrated Plasma and Neuroimaging Biomarkers Associated with Motor and Cognition Severity in Parkinson’s Disease. J. Parkinson’s Dis..

[43] Kuhle J., Barro C., Andreasson U., Derfuss T., Lindberg R., Sandelius Å., Liman V., Norgren N., Blennow K., Zetterberg H. (2016). Comparison of three analytical platforms for quantification of the neurofilament light chain in blood samples: ELISA, electrochemiluminescence immunoassay and Simoa. Clin. Chem. Lab. Med..

[44] Das S., Dewit N., Jacobs D., Pijnenburg Y.A.L., In ‘t Veld S.G.J.G., Coppens S., Quaglia M., Hirtz C., Teunissen C.E., Vanmechelen E. (2022). A Novel Neurofilament Light Chain ELISA Validated in Patients with Alzheimer’s Disease, Frontotemporal Dementia, and Subjective Cognitive Decline, and the Evaluation of Candidate Proteins for Immunoassay Calibration. Int. J. Mol. Sci..

